# Living Bacteria Directly Embedded into Electrospun Nanofibers: Design of New Anode for Bio-Electrochemical Systems

**DOI:** 10.3390/nano11113088

**Published:** 2021-11-16

**Authors:** Giulia Massaglia, Adriano Sacco, Angelica Chiodoni, Candido Fabrizio Pirri, Marzia Quaglio

**Affiliations:** 1 Department of Applied Science and Technology (DISAT), Politecnico di Torino, Corso Duca degli Abruzzi 24, 10129 Torino, Italy; fabrizio.pirri@polito.it; 2 Center for Sustainable Future Technologies (CSFT)@Polito, Istituto Italiano di Tecnologia, Environment Park, Building B2 Via Livorno 60, 10144 Torino, Italy; adriano.sacco@iit.it (A.S.); angelica.chiodoni@iit.it (A.C.)

**Keywords:** electrospinning, nanofibers, bio-composite nanofibers, microbial fuel cells

## Abstract

The aim of this work is the optimization of electrospun polymeric nanofibers as an ideal reservoir of mixed electroactive consortia suitable to be used as anodes in Single Chamber Microbial Fuel Cells (SCMFCs). To reach this goal the microorganisms are directly embedded into properly designed nanofibers during the electrospinning process, obtaining so called nanofiber-based bio-composite (bio-NFs). This research approach allowed for the designing of an advanced nanostructured scaffold, able to block and store the living microorganisms inside the nanofibers and release them only after exposure to water-based solutions and electrolytes. To reach this goal, a water-based polymeric solution, containing 5 wt% of polyethylene oxide (PEO) and 10 wt% of environmental microorganisms, is used as the initial polymeric solution for the electrospinning process. PEO is selected as the water-soluble polymer to ensure the formation of nanofiber mats offering features of biocompatibility for bacteria proliferation, environment-friendliness and, high ionic conductivity. In the present work, bio-NFs, based on living microorganisms directly encapsulated into the PEO nanofiber mats, were analyzed and compared to PEO-NFs made of PEO only. Scanning electron microscopy allowed researchers to confirm the rise of a typical morphology for bio-NFs, evidencing the microorganisms’ distribution inside them, as confirmed by fluorescence optical microscopy. Moreover, the latter technique, combined with optical density measurements, allowed for demonstrating that after electrospinning, the processed microorganisms preserved their proliferation capability, and their metabolic activity after exposure to the water-based electrolyte. To demonstrate that the energy-production functionality of exo-electrogenic microorganisms was preserved after the electrospinning process, the novel designed nanomaterials, were directly deposited onto carbon paper (CP), and were applied as anode electrodes in Single Chamber Microbial Fuel Cells (SCMFCs). It was possible to appreciate that the maximum power density reached by bio-NFs, which resulted in being double of the ones achieved with PEO-NFs and bare CP. SCMFCs with bio-NFs applied as anodic electrodes reached a current density value, close to (250 ± 5.2) mA m^−2^, which resulted in being stable over time and was comparable with the one obtained with carbon-based electrode, thus confirming the good performance of the whole device.

## 1. Introduction

Sustainable green transition is the main topic behind the European Green Deal [1,2], which aims to achieve climate neutrality by 2050 [1,2]. In this scenario, the use of renewable energy sources has gained great interest during last years since they played a pivotal role in reducing greenhouse gas emissions, guaranteeing the diversification of energy supplies and to minimizing the dependency on fossil fuel markets, such as oil and gas. Among the most diffuse renewable energy sources, such as wind, solar, hydro- and- geo- thermal biomass, a great interest has arisen to the development of alternative green systems able to combine energy production with biotechnological approaches for water treatment, bioremediation and sensing [3,4,5,6].

Microbial Fuel Cells (MFCs) belong to that class of systems at the crossroad between energy and biotechnology. They are bio-electrochemical systems successfully used to combine energy production with several treatments thanks to the great potential and versatility of whole exo-electrogenic microorganisms [7,8]. Basically, MFCs directly convert the chemical energy contained in organic matter, known as fuels, into electrical energy by the metabolic activity of exo-electrogenic microorganisms. These microorganisms, indeed, are able to catalyze the oxidation reaction of fuel producing an electrical current by transfer of produced electrons to the anode surface. Subsequently, the electrons flow through an applied external load from the anode compartment to the cathodic one, where the terminal electron acceptor (TEA), usually oxygen, is then reduced [8,9,10]. Into the anode compartment of these devices, the microorganisms proliferate in intimate contact with the anode surface, ensuring thus the formation of a biofilm, which results in being pivotal to guarantee energy conversion and the electron transfer process. The electrical power output of MFCs is typically low, and it is currently hindering the marketability of MFCs [11]. For this reason, several works in the literature focused their attention on the optimization of anode surface [12,13].

Indeed, since the electrical power output is strictly correlated to the electroactivity of these bacteria, the optimization of the anodic electrode and its interface with the bacterial biofilm plays a pivotal role in determining the electrical performance of the MFC devices [12,13,14,15,16,17,18,19,20,21,22,23,24,25,26,27]. Anode porosity has a crucial importance to increase the effective surface available for bacteria adhesion and proliferation, thus improving electron transfer to the surface [12,13].

During the last decades, many researchers demonstrated that the modification of carbon-based materials, commonly employed as anode electrodes, by applying polymeric films on their surface is an effective approach to improve the contact among bacteria and anodes in MFCs [14,15,16,17], thus enhancing the electron transfer at that interface. Furthermore, many works in the literature focused their attention on better understanding the complexity of biofilms and their unique features with the principal purpose of creating synthetic ones, optimized for different biotechnological applications, such as environmental remediation [18,19], fermentation reactors [20], particle biofilm reactors [21] and microbial fuel cells [22,23]. In recent years, the development of systems to encapsulate exo-electrogenic bacteria to be used in MFCs attracted a great deal of interest, aimed to reduce the effects of environmental parameters on the bio-electrocatalytic activity of electroactive microorganisms on [23,24,25,26,27]. Chen et al. encapsulated a pre-grown living electroactive biofilm with a poly (vinyl alcohol) hydrogel, demonstrating that the microorganisms kept unchanged their bio-electroactivity in MFCs, moreover improving their stability also in alkaline conditions [26]. Sanchez et al. encapsulated the electroactive bacteria Shewanella Oneidensis into core-shell nanofibers, significantly improving the final power output of MFCs containing this bioanodes [27]. In the present work, nanofiber-based bio-composites (bio-NFs) are proposed as anodes in MFCs where, mixed electroactive consortia are directly embedded into polyethylene oxide (PEO) nanofibers.

**Scheme 1:**
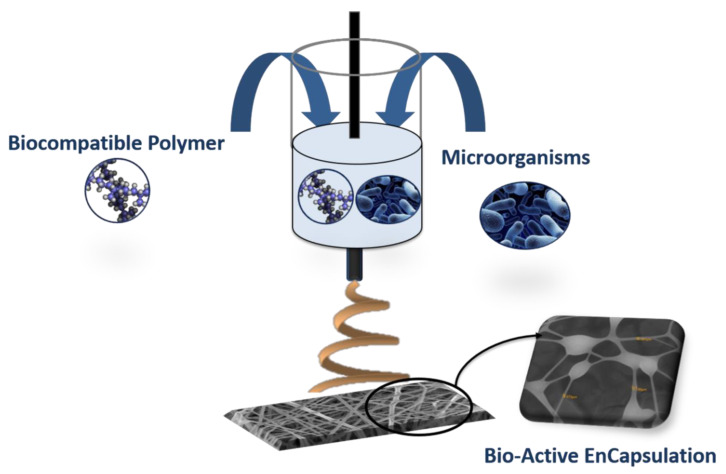
Sketch of electrospun nanofiber-based bio-composite obtained by starting from a water-based polymeric solution, containing 5wt% of polyethylene oxide (PEO, Mw = 600 kDa, purchased from Sigma Aldrich) and 10 wt% of environmental microorganisms, is selected as initial polymeric solution for the electrospinning process.

The aim of the present work is to optimize electrospun polymeric nanofibers as a reservoir for mixed microorganisms, in so-called nanofiber-based bio-composite (bio-NFs). This research approach allows for the designing of an advanced 3D nanostructured scaffold, able to block the living microorganisms inside the nanofibers, and to release them selectively only after exposure of the water-soluble polymer to the water-based electrolyte. To this purpose, the electrospinning process was selected, since in the last years many works in the literature demonstrated its suitability to develop even complex nanostructures, suitable for encapsulating active elements for functional applications [23,24,27,28,29]. Electrospinning offers the possibility to design several porous arrangements of nanofibers by blending different polymers in single-fluid processes, as well as fabricating more complex systems made of coaxial or tri-axial NFs [28]. However, a very limited number of publications can be found for loading living bacteria for application in bio-electrochemical systems as MFCs [23,24,27], none of them investigating the bio-electroactive behavior of mixed consortia after encapsulation. To this purpose, in the present work, a water-based polymeric solution, containing 5 wt% of polyethylene oxide (PEO, M_w_ = 600 kDa, purchased from Sigma Aldrich, Saint Louis, MO, USA) and 10 wt% of environmental microorganisms, is selected as initial polymeric solution for the electrospinning process, as represented in the Scheme 1. PEO is selected as the polymer to ensure the formation of biocompatible and environmentally friendly nanofiber mats, well-suited for bacteria proliferation and, moreover, able to act as a good polyelectrolyte, and able to provide high ion’s mobility and rate of transfer. Moreover, it is important to highlight that the presence of PEO, which is a water-soluble polymer, allowed the spontaneous transformation of nanofibers into a soft hydrogel layer after exposure to the water-based electrolyte, leading thus to improve ion mobility, diffusion and transfer rate, actually working as a quasi-solid polymer electrolyte in this electrochemical application [27,28,29,30]. Electrospinning process was performed to provide two different nanofiber mats: (i) bio-NFs where electroactive bacteria are directly encapsulated into them; (ii) PEO-NFs based on only PEO, used as reference material. During the process, a high positive voltage equal to 10 kV, granting thus the formation of final nanofiber mats without affect the living microorganisms. Nanofiber mats were directly collected onto a carbon-based material, according to Electrospinning-on-Electrode process, a binder-free method for nanofiber assembly onto an electrode [31].

Electrospinning-on-Electrode assembly ensure the arrangements of nanofibers with an optimized electrochemical interface, thus leading to achievement of an improved biofilm formation, needed to enhance the electrical power output of MFCs. Morphological properties of all nanomaterials were analyzed with Field Effect scanning Electron Microscopy (FESEM, ZEISS Merlin, 5–10 kV), while a fluorescence microscopy analysis, ((Nikon ECLIPSE Ni, Japan), is implemented to demonstrate the preserved microorganisms’ vitality after the electrospinning process. To demonstrate the preservation of their metabolic activity and their capability to proliferate also after the electrospinning process, optical density measurements (OD, LAMBDS 850+ UV/Vis Spectrophotometer) was employed. Optical density (OD) of the culture is employed to estimate the growth and metabolic activity of the cells. Indeed, OD is defined as a logarithmic function wherein a higher number of light absorptions means higher bacterial growth.

Subsequently, these bio-NFs were applied as anodic electrodes in bio-electrochemical devices (i.e., Single Chamber Microbial Fuel Cells, SCMFCs). In particular, all designed nanomaterials, directly deposited onto carbon paper and bare carbon paper (CP), were applied as anode electrodes in SCMFCs. It was possible to appreciate that maximum power density reached by bio-NFs resulted to be double of the ones achieved with PEO-NFs and bare CP. SCMFCs with bio-NFs applied as anodic electrodes reached a current density value, close to (250 ± 5.2) mA m^−2^, which resulted to be stable over time, thereby confirming the good performance of the whole device.

## 2. Materials and Methods

### 2.1. Materials and Nanofibers Synthesis

Electroactive bacteria comprise a mixed environmental sample derived from sea-water sediment. A water- based polymeric solution with 5 wt% of Polyethylene oxide (PEO, Mw = 600 kDa, purchased from Sigma Aldrich, Saint Louis, MO, USA) was continuously stirred for one day at room temperature. Moreover, to obtain nanofiber-based bio-composite (bio-NFs), a water-based solution containing 10 wt% of electroactive bacteria with respect to PEO, was successively added to obtain the final polymeric solution. The mixed culture of bacteria was dissolved into the electrolyte, based on sodium acetate (C_2_H_3_NaO_2_), ammonium chloride (0.31 g L^−1^ of NH_4_Cl) used as nitrogen source to aid the bacteria growth, and on phosphate buffer solution (PBS) able to maintain a neutral pH. As already reported in our previous works [32,33,34] NANON 01A machine from MECC Co. Ltd., Fukuoka, Japan was used to implement the electrospinning process and obtain final dried nanofiber mats. The polymeric solution was loaded into a syringe and nanofibers were obtained by applying a high positive voltage equal to 10 kV, a flow rate of 0.3 mL h^−1^ and a working distance of 15 cm. These process parameters were selected to guarantee the nanofiber mats deposition but not affecting the living microorganisms into the initial polymeric solution. All nanofiber mats were directly collected onto a carbon-based material (carbon paper, CP, purchased from Fuel Cell Earth, Woburn, MA, USA), commonly used as anode electrode in many bio-electrochemical systems, ensuring a binder free electrospinning-on-electrode assembly [31]. Each electrospinning process lasted 1 h, allowing the depositing of about 1 mL of microorganisms containing solution over each electrode. This process time was selected to allow the use of an inoculum source equal to that used in standard conditions, as described in our previous work [33].

### 2.2. Characterizations and Measurements

The morphological properties of all nanomaterials were analyzed with Field Effect Scanning Electron Microscope (FESEM, ZEISS Merlin) operating between 5 and 10 kV. Since one of the main aims of the present work is based on the demonstration that the bacteria, embedded into nanofibers, are able to preserve their vitality, in terms of metabolic activity and their proliferation, optical density (OD) measurements were carried out by means of a LAMBDA 850+ UV/Vis Spectrophotometer. Indeed, OD measurement estimated the growth and metabolic activity of bacteria. Previously all samples, bio-NFs, PEO-NFs and bare CP were put in a Falcon Conical Tubes containing the electrolyte solution. Electrolyte solution is a water-based solution containing all chemical compounds suitable for the metabolic activity of electroactive bacteria, such as 1 g L^−1^ sodium acetate (C_2_H_3_NaO_2_), ammonium chloride (0.31 g L^−1^ of NH_4_Cl), used as nitrogen source to favor bacterial growth and phosphate buffer solution to guarantee a neutral pH. Moreover, for PEO-NFs and bare CP, some amount of microorganisms, embedded into bio-NFs, were added. All samples were left for 15 days. To demonstrate the preserved microorganisms’ vitality after the electrospinning process, optical fluorescence microscopy was used, specifically, Fluorescent microscopy (Nikon ECLIPSE Ni, Amsterdam, Netherlands) was employed. Prior to imaging analysis, bio-NFs were stained using a LIVE/DEAD BacLight Bacterial Viability Kit (Invitrogen, Waltham, MA, USA) [35]. Briefly, bio-NFs mats were stained for 20 min by employing the live/dead bacterial kit, and then washed in sterile PBS twice to eliminate the dye excess. Imaging analysis was performed using NIS Elements Image Software.

### 2.3. MFCs Architecture and Configuration

Anode electrodes were designed to test the electroactive behavior of the encapsulated microorganisms in open-air cathode Single Chamber Microbial Fuel Cells. In the present work, as described in our previous works, the SCMFCs were designed and obtained by 3D OBJECT printing [36]. In particular, SCMFCs are composed by three compartments, the anodic part, the intermediate one and cathode part. These devices are membrane less SCMFCs, where the electrolyte was in common with anode and cathode compartments. The total internal volume of SCMFCs is 12.5 mL. Both anodes and cathodes electrodes showed carbon-based material (carbon paper CP, purchased from FUEL CELL EARTH, Woburn, MA, USA), employed as carbon backbone to ensure the electron transfer produced and released by microorganisms. These electrodes, moreover, showed a geometric area equal to 5.76 cm^2^. Furthermore, in the present work, three different anode electrodes were investigated and compared: (i) bio-NFs engineered to obtain final nanofibers where the microorganisms are directly encapsulated into them; (ii) PEO-NFs designed by the direct deposition of PEO nanofiber mats on carbon-based electrode, employed as reference material; (iii) the carbon-based material (CP) used as control. As reported in our previous work [36], to enhance the oxygen reduction reaction, the cathode electrode was modified properly, presenting a gas diffusion layers based on polytetrafluoroethylene (PTFE) on the outer side and a catalyst layer (CLs) based on Platinum (Pt/C 0.5 mg cm^−2^) and Nafion (5 wt% Nafion, from Sigma Aldrich, Saint Louis, MO, USA) as a binder [33]. Titanium wires (Goodfellow Cambridge Limited) were fixed onto anode and cathode electrodes through a conductive paste made of carbon cement (Leit-C Cement). A water-based electrolyte solution was based on sodium acetate (C_2_H_3_NaO_2_) used as carbon-energy source with a concentration of 1 g L^−1^, together with other compounds that are able to ensure the optimal operation of SCMFCs. All these compounds are based on ammonium chloride (0.31 g L^−1^ of NH_4_Cl) used as nitrogen source to support the bacteria growth, and phosphate buffer solution (PBS) able to maintain a neutral pH (constituted by 0.13 g L^−1^ of potassium chloride, 4.28 g L^−1^ of sodium phosphate dibasic and 2.45 g L^−1^ of sodium phosphate monobasic monohydrate). The solution was autoclaved prior to use. SCMFCs were inoculated with a mixed culture of bacteria from a seawater sediment, which contained electroactive bacteria able to catalyze the organic matter, transducing the chemical energy contained into the fuel directly into electrical energy. During these experiments, sodium acetate has been selected as the carbon-energy source. Sodium acetate ensures the production of a number of electrons equal to eight, after full oxidation by the microorganisms [37]. All experiments are conducted in triplicate. Differently from all SCMFCs, contained CP and PEO-NFs as anode electrodes, the SCMFCs with bio-NFs as anode electrodes were no inoculated with a mixed culture of bacteria from sea-water sediment. In this way, it was possible to demonstrate that all embedded microorganisms were able to proliferate and catalyze the oxidation reaction of sodium acetate, thus ensuring the electrons’ transfer rate from bacteria to anode electrodes. Anodes and cathodes of SCMFCs were connected to a multichannel data acquisition unit (Agilent 34972A), and two different values of external resistance were applied. The whole experiments can be divided in two different phases: the first one, known as acclimation phase, was provided by applying an external load equal to 500 Ω, with the main purpose to evaluate the repeatability of voltage trends. The second phase, on the contrary, was carried out by using a higher external resistance of 1 kΩ to define the overall SCMFCs’ performance. During whole experiment, a fed batch mode was defined, meaning that the electrolyte was replaced with the new one when the voltage drops results to be close to 0 mV. Polarization curves are defined through linear sweep voltammetry characterizations (LSV), performed by using VSP, Biologic Potentiostat. LSV characterizations by implementing a voltage range from open circuit to a short circuit with a rate of 0.1 mV s-1. Internal resistances of the SCMFCs have been evaluated through the Nyquist plots using electrochemical impedance spectroscopy (EIS). EIS has been conducted at Open Circuit Voltage (OCV) over the range of frequency between 150 kHz and 200 mHz with a sinusoidal signal with an amplitude of 25 mV.

## 3. Results and Discussion

### 3.1. Morphological and Biological Characterizations

**Figure 1:**
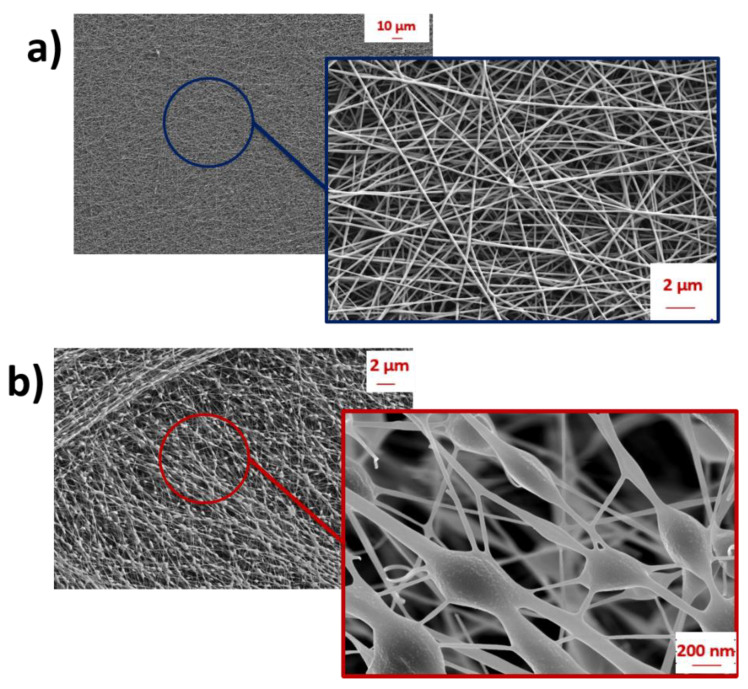
(**a**) FESEM images representing the uniform distribution of PEO-NFs onto carbon-based material without defects, as beads, drops, occurred during the electrospinning process. In the blue box, the ordered distribution of PEO-NFs onto the conductive CP is highlighted; (**b**) FESEM images reporting the presence of microorganisms directly encapsulated into nanofiber mats.

A uniform distribution of PEO-NFs without the presence of several defects, such as beads, drops that can be occurred during the electrospinning process, is confirmed by FESEM characterizations (see Figure 1a). This consideration allowed confirming how the presence of some protrusions along the main symmetric axes of nanofibers was allocated to the microorganisms’ distribution inside them, as highlighted in Figure 1b. No presence of several defects for both of the nanostructures, as PEO-NFs and bio-NFs, allowed confirmation of how the initial polymeric solution, developed for the electrospinning process, resulted in being suitable and was optimized to obtain of a uniform distribution of nanofiber mats. Moreover, as deeply reported in our previous works [25], the presence of carbon-based materials as substrate, where nanofiber mats were directly collected, affect the distribution of nanofibers themselves, leading thereby to induce an ordered arrangement of PEO-NFs onto CP materials. In line with this phenomenon, final nanofibers mats showed a high surface area to volume ratio with an improved porosity that enhance the properties of anode electrodes, as also confirmed by many works in the literature [12,13].

**Figure 2:**
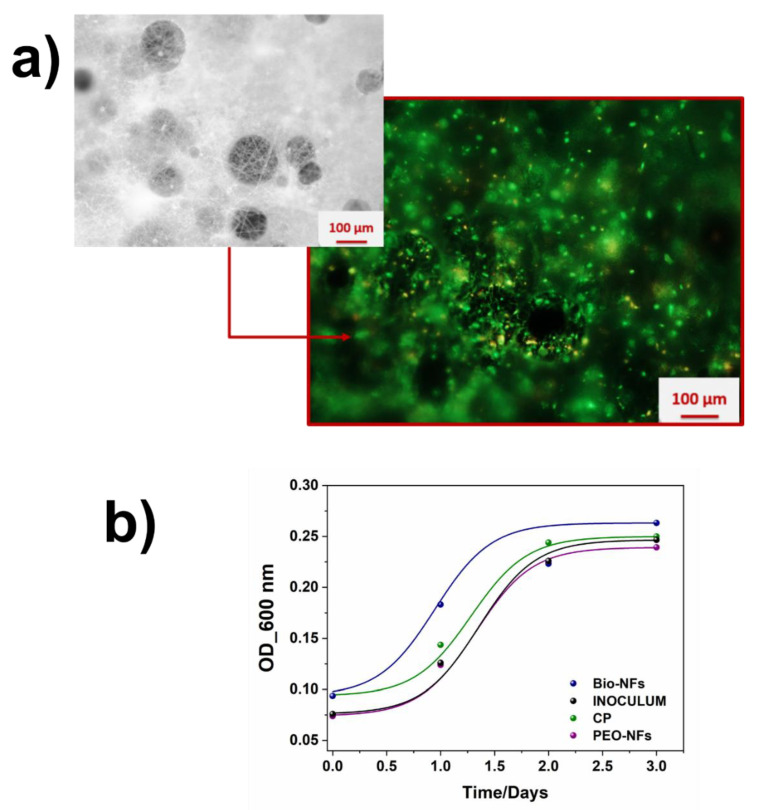
(**a**) Optical fluorescence characterizations of bio-NFs. In the red box, the presence of a certain amount of green spots confirmed the preservation of bacterial vitality after the electrospinning process; (**b**) Optical density (OD) measurement.

The presence of living bacteria directly embedded into nanofibers was proved by optical fluorescence characterizations and OD measurements conducted on bio-NFs. Fluorescence images showed a higher number of green spots than the one of red spots, as reported in the Figure 2a. Green spots represented all living microorganisms embedded into nanofibers, while red spots highlighted bacteria that had died. These results, shown above, demonstrated that the electrospinning process was not detrimental to the microorganisms. With the main purpose to define the capability of bacteria to resume their metabolic activity and proliferation when bio-NFs were in contact with the electrolyte, OD measurements were carried out. As reported in Figure 2b, light absorption for bio-NFs results in being higher than the ones obtained when all other materials were tested, such PEO-NFs, CP and only inoculum solutions used as reference sample. Starting from these characterizations, furthermore, it was possible to demonstrate two important hypotheses. The first one is strictly correlated with the capability of bacteria, embedded into nanofibers, to resume their metabolic activity and proliferation also after the electrospinning process. The second important result, confirmed by OD measurements, is related to the fact that the transformation of nanofibers into a hydrogel material, due to the presence of a water-soluble polymer as PEO, does not lead the release of chemical substances, which may be toxic to bacteria. Finally, bio-NFs can be considered as a reservoir for the microorganisms, and are able to preserve the bacteria from environmental variations.

### 3.2. SCMFCs Performance

**Figure 3:**
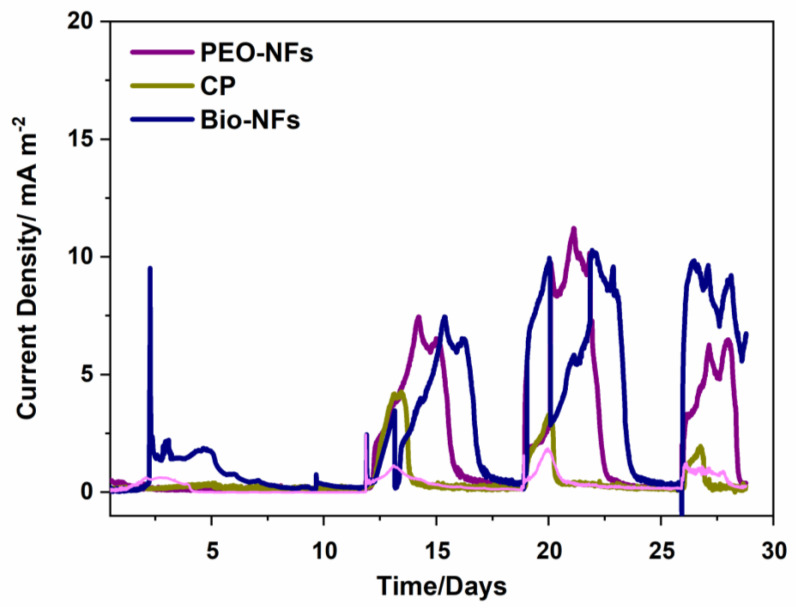
Current density produced during the acclimation phase when an external load of 500 Ω was applied: bio-NFs (blue line) PEO-NFs (purple line) and carbon-based material used as reference electrode (green line).

In the present work, the development of delivery systems for electroactive microorganisms in SCMFCs is an important issue to protect them from the environmental variations, thereby achieving the preservation of the bio electrocatalytic activity of electrodes, improving also their overall performance. In the present work, with the main aim to demonstrate the preservation of metabolic activity and proliferation capability of bacteria embedded into nanofibers, all designed nanomaterials were applied as anode electrodes in SCMFCs. Bio-NFs and PEO-NFs were directly deposited onto carbon-based material, while bare carbon-based material (CP) was used as reference material. The whole experiment can be divided in two phases: (i) acclimation phase; (ii) second phase where overall SCMFCs performance were evaluated. In particular, during the first phase of the experiments, known as acclimation phase, an external resistance close to 500 Ω were applied, to induce the biofilm formation onto electrodes. All SCMFCs, which presented PEO-NFs and CP as anode electrodes, were inoculated with a mixed culture derived from a sea-water sediment. Differently SCMFCs that showed bio-NFs as anode electrodes were filled with only electrolyte, without the addition of further microorganisms. The duration time of this acclimation phase is close to 1 month. As represented in Figure 3, it is possible to highlight how the currrent denisty reached by bio-NFs resulted in being almost double the one obtained with PEO-NFs and CP. This results allowed confirmation of how the development of delivery systems for electroactvie bacteria, able to protect them from environmental variations, affected and improved biofilm’s formation, thus reflecting onto the overall device performance. Indeed, when bio-NFs were in contact with water-based electroyte, the presence of PEO, which is a water-soluble polymer, granted the transformation of nanofibers into a hydrogel material, thereby ensuring a high ion mobility, a high ion diffusion and transfer rate, also avoiding the release of chemical substances that can be toxic for biofilm. In line with several works, a good electrical output produced by SCMFCs was achieved, demonstrating a stable and good bacteria proliferation on anode electrodes (see Figure 3).

**Figure 4:**
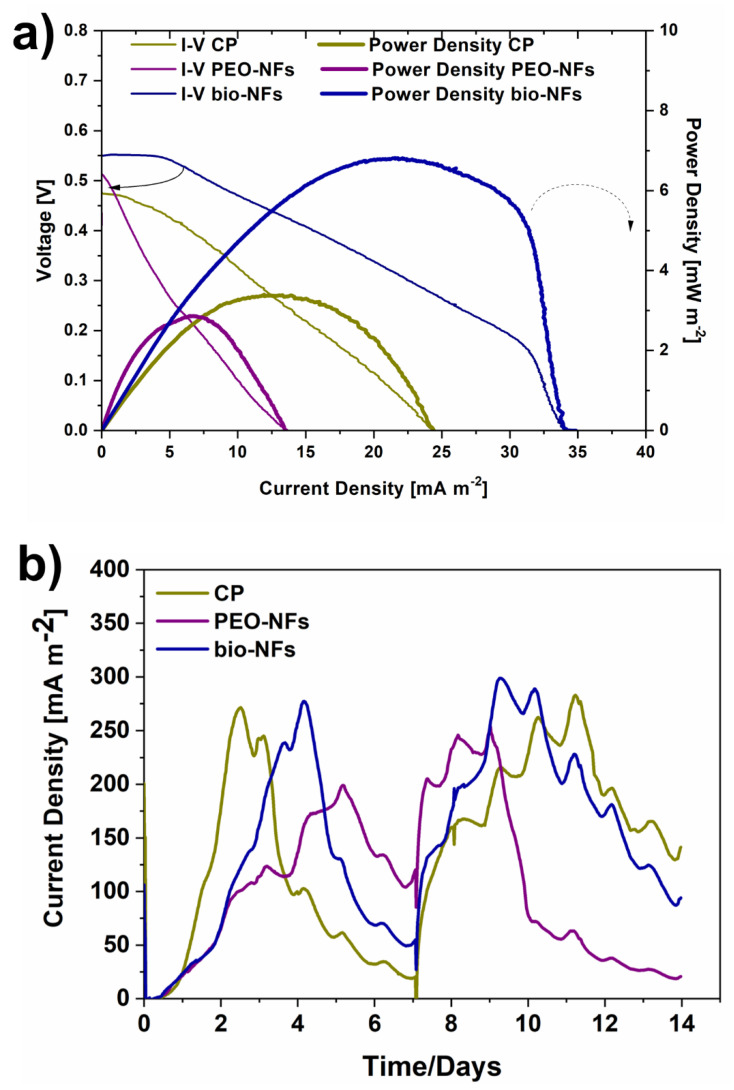
(**a**) Polarization curves obtained by LSV characterization. Potential vs. current density curves (left axis, dash lines) and power density vs. current density (right axis, straight lines) curves of SCMFCs with bio-NFs nanofibers, PEO-NFs and bare carbon-based material CP as reference materials (**b**) Comparison of current density trend of SCMFCs with bio-NFs nanofibers, PEO-NFs and bare carbon-based material CP.

The overall performance reached with bio-NFs as anode electrodes were compared with a reference SCMFCs, which showed a bare CP and PEO-NFs as anode electrode. In Figure 4a), obtained polarization curves, were represented. It was possible to appreciate that the maximum power density with bio-NFs resulted in being double of the ones obtained with PEO-NFs and CP, applied as anode electrodes, leading thus to demonstrate how nanostructured bio-NFs granted the preservation of vitality of microorganisms and consequently an optimized biofilm formation with an improved interface between biofilm and anode surface. Moreover, SCMFCs with bio-NFs nanofibers reached a higher short circuit current, close to (34.87 ± 0.54) mA m^−2^ with respect to values reached with PEO-NFs (equal to 13.6 ± 0.5 mA m^−2^) and with bare carbon-based material CP (equal to 25.1 ± 0.5 mA m^−2^). All current density values were obtained by normalizing the measured current output over time with the geometric area of SCMFCs, close to 5.76 cm^2^. As represented in Figure 4b, bio-NFs reached a high current density value, close to 250 ± 5.2 mA m^−2^, which result in being stable over time, thus leading to confirmation of the good interaction between the biofilm proliferated onto anode electrodes. Since these devices presented formally identical cathode electrodes, it was possible to address all variations to the anode electrodes, demonstrating the improvement achieved when bio-NFs were applied as anode electrodes. The design of advanced nanostructured scaffold, able to block the living microorganisms inside the nanofibers, thereby leading to release them when the polymer was in contact with water since the latter polymer is water-soluble, played a pivotal role to enhance the overall SCMFCs performance.

To better appreciate the results achieved in this work, it is worth noting that a mixed community from seawater samples has been used. This choice is important in improving the robustness of the bio-electrochemical systems with respect to those running with pure electroactive culture. Sanchez et al. published an interesting work fabricating complex co-axial nanofibers encapsulating a pure culture of electroactive Shewanella Oneidensis [26], obtaining a current output equal to 315 mA/m^2^. In the present work, the bioanodes obtained using the mixed culture from sediments encapsulated in the water-soluble bio-NFs reached a current density of 250 mA/m^2^. This result is in line with the finding from Sanchez and colleagues, but it is worth noting that the here proposed bioanode is quite simpler to be fabricated, since it requires one polymeric solution only with most simple electrospinning setup. Moreover, it has a huge potential to be a useful approach for other applications involving other treatments to be combined with energy conversion, first of all, environmental sensing [38].

### 3.3. Electrochemical Impedance Spectroscopy Results

**Figure 5:**
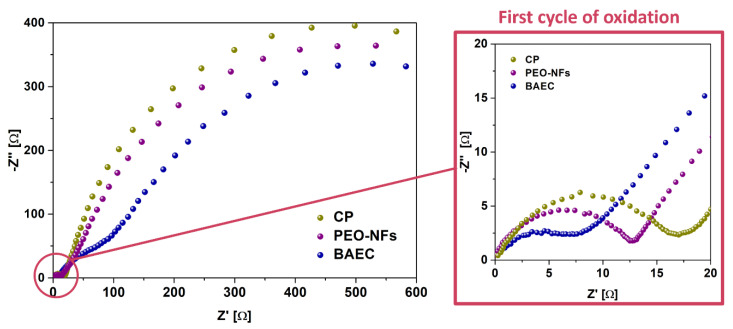
Impedance spectra of CP/PEO NFs (black dot and line representing the experimental data and their fitting) and CP anode (green dot and line).

EIS has been performed to define impedance behavior with great attention given to the internal resistance correlated to the charge transfer of all different anode electrodes R_ct_ [39,40,41]. By analyzing the Nyquist plot represented in Figure 5, it can be observed that bio-NFs showed a lower charge transfer resistance, as reported in the magnitude of the first cycle obtained into EIS characterization, sketched on the right side of Figure 5. A lower charge transfer resistance, thus, leads to confirmation of the capability of microorganisms to proliferate in a correct way onto carbon-based material and to release the electrons outside from their cells towards carbon paper. On the contrary a higher charge resistance of PEO without microorganisms, highlighted by blue curve can be ascribed to the insulator properties of PEO.

## 4. Conclusions

In the present work, nanofiber-based bio-composite (bio-NFs) were proposed as anodes electrodes in MFCs where electroactive bacteria are directly embedded into Polyethylene oxide (PEO) nanofibers. We proposed the optimization of electrospun polymeric nanofibers as a reservoir of bacteria, which are directly embedded into the nanofibers, obtained so called nanofiber-based bio-composite (bio-NFs). This research approach allowed for the designing of an advanced nanostructured scaffold, able to block the living microorganisms inside the nanofibers, thus leading to release them when the polymer was in contact with water since the latter polymer is water-soluble. Indeed, as confirmed by OD measurements and optical fluorescence, the capability of microbial proliferation and metabolic activity resulted in being preserved for those microorganisms encapsulated into nanofibers. Thanks to the presence of nanofibers, which act as reservoir for bacteria, the microorganisms themselves were protected by environmental variations, thereby leading to the maintaining of the bio-electrocatalytic properties, exploited when the nanofiber mats were in contact with electrolyte solution. We designed and engineered an anode electrode that showed all the properties needed to improve the overall SCMFCs performance. Future-oriented applications of this nanostructured scaffold, able to block and store up to water/electrolyte exposure the living microorganisms inside the nanofibers, can be ascribed to the development of optimized anode electrodes applied in bio-electrochemical devices in which mixed consortia can be used to combine energy production and different as environmental remediation, water treatment and environmental sensing.

## Data Availability

The data presented in this study are available on request from the corresponding author.
